# Reliability of the Seated Unilateral Cable Row and Strength Differences Between Dominant and Non-Dominant Sides in Young Athletes

**DOI:** 10.3390/jfmk10040390

**Published:** 2025-10-07

**Authors:** Ángela Rodríguez-Perea, Helena Vila, Carmen Ferragut, Daniel Jerez-Mayorga, Luis Javier Chirosa Ríos, Oscar García-García, Virginia Serrano-Gómez

**Affiliations:** 1 Department of Physical Education and Sport, Faculty of Physical Activity and Sports Sciences, Universidad de León, 24007 León, Spain; arodrp@unileon.es; 2 Strength & Conditioning Laboratory, CTS-642 Research Group, Department Physical Education and Sports, Faculty of Sport Sciences, University of Granada, 18071 Granada, Spain; djerezmayorga@ugr.es (D.J.-M.); lchirosa@ugr.es (L.J.C.R.); 3 Department of Sports’ Special Didactics, University of Vigo, 36005 Pontevedra, Spain; oscargarcia@uvigo.es; 4 Laboratory of Sports Performance, Physical Condition and Wellness, Faculty of Education and Sports, University of Vigo, 36310 Pontevedra, Spain; 5 Department of Biomedical Sciences, Faculty of Medicine and Science Health, University of Alcala, 28801 Alcalá de Henares, Spain; carmen.ferragut@uah.es; 6 Exercise and Rehabilitation Sciences Institute, Faculty of Rehabilitation Sciences, School of Physical Therapy, Universidad Andres Bello, Santiago 7591538, Chile

**Keywords:** asymmetries, isokinetic, performance, imbalance

## Abstract

**Background:** Muscle strength asymmetries between limbs are common in physically active populations and may influence performance and injury risk. This study aimed to: (i) analyze the reliability of the seated unilateral cable row exercise using a functional electromechanical dynamometer (FEMD) and to examine differences in reliability between sides and contraction types; (ii) investigate the relationship between the dominant and non-dominant sides, as well as between the dynamic and static force production of the back muscles; and (iii) quantify force output and assess interlimb asymmetries. **Methods:** Twenty-nine young physically active athletes completed two sets of four repetitions of a seated unilateral cable row at 0.30 m·s^−1^ using the FEMD, followed by a 6-s isometric contraction. Two testing sessions were conducted seven days apart. Reliability was assessed using paired *t*-tests, the effect size, the coefficient of variation (CV), the standard error of measurement, and the intraclass correlation coefficient (ICC), with 95% confidence intervals. **Results:** Peak and average force values showed very high to extremely high relative reliability (ICC = 0.86–0.96) and acceptable absolute reliability (CV ≈ 10%). Differences between dominant and non-dominant sides varied depending on contraction type. While group-level asymmetries did not exceed 10%, individual analysis revealed that 14%, 32%, and 7% of participants had asymmetries greater than 15% in isometric, concentric, and eccentric force, respectively. **Conclusions:** This test demonstrates strong reliability and provides a practical method for assessing upper limb asymmetries in physically active individuals, with potential applications in performance monitoring and injury prevention.

## 1. Introduction

Muscle force is a well-established indicator of physical health and a key predictor of exercise capacity. In fact, the ability to generate force is closely associated with the risk of musculoskeletal injuries [1]. Within this context, the back musculature plays a critical role, as adequate force production is essential for both injury prevention and performance enhancement in athletes and the general population [2,3].

Traditional training and assessment methods have frequently relied on bilateral movement (e.g., deadlifts or rows) [4,5]. However, these approaches may conceal underlying muscular imbalances, limiting their capacity to provide a comprehensive evaluation. A central concern in sports science is the recognition and management of interlimb asymmetries, defined as differences in strength, power, or range of motion between the dominant and non-dominant sides [6]. Such imbalances are particularly prevalent in unilateral sports, where repetitive loading of one side increases the risk of performance deficits and injury [7,8]. While bilateral training may obscure these asymmetries, unilateral training provides a targeted strategy for their identification and reduction. Consequently, assessing not only overall force output but also interlimb asymmetries has become a key objective in strength and conditioning.

Muscle force can be assessed through various approaches, ranging from traditional tools to more advanced technologies, including isoinertial systems [9] and functional electromechanical dynamometry (FEMD) [10]. Recent advances in electromechanical devices have enabled the assessment of multiple contraction modes, including isometric and isokinetic [11]. Earlier evaluations relied heavily on isokinetic devices, which are costly and often lack ecological validity for sport-specific movements. Moreover, these devices typically isolate individual muscle actions—for instance, in the case of the back musculature, they primarily assess extensor muscles [12]. Assessments of rowing-related movements have traditionally employed free weights or isometric tests using linear encoders or load cells, which predominantly capture the concentric phase [13,14]. Despite these developments, a significant methodological gap persists in the accurate evaluation of unilateral, multi-joint back exercises, which are fundamental in both athletic preparation and rehabilitation.

Therefore, the aims of this study were to (i) analyze the reliability of the seated unilateral cable row exercise using a FEMD and to examine differences in reliability between sides and contraction types; (ii) investigate the relationship between the dominant and non-dominant sides, as well as between dynamic and static force production of the back muscles; and (iii) quantify force output and assess interlimb asymmetries. We hypothesized that the seated unilateral cable row exercise would exhibit high absolute and relative reliability, that force production between the dominant and non-dominant sides—as well as between dynamic and static contractions—would show strong correlations, and that the dominant side would demonstrate greater force output.

## 2. Materials and Methods

### 2.1. Participants

Twenty-nine young, physically active athletes (21% female; 21.38 ± 1.29 years, 1.74 ± 0.1 m, 72.8 ± 14.4 kg and 23.97 ± 2.86 kg/m^2^) participated in this study. Participants were recruited from the university population through informational posters displayed across campus and targeted email invitations sent to students and staff. Eligibility criteria included being physically active, free from musculoskeletal injuries, and having no previous experience with FEMD. Physical activity levels were verified using the International Physical Activity Questionnaire (IPAQ). All participants were informed about the nature, aims, and risks associated with the experimental procedure before they provided written consent to participate. The study protocol was approved by the Research Ethics Committee of the University of Granada (approval code: 2560/CEIH/2022) and was conducted in accordance with the ethical guidelines of the Declaration of Helsinki.

### 2.2. Procedures

A repeated-measures design was used to evaluate the seated unilateral cable row exercise using a FEMD (Symotech, Granada, Spain) [11]. Participants attended the laboratory on two separate occasions. During the first visit, anthropometric measurements were recorded, and participants completed the IPAQ. They were then familiarized with the testing procedures before performing the initial seated unilateral cable row exercise. The retest session was scheduled one week after the initial assessment to ensure full recovery and to minimize potential learning or fatigue effects. The retest protocol mirrored the initial session exactly, including the warm-up, equipment setup, and testing procedures, to ensure consistency and reliability of the measurements. All assessments were conducted by the same evaluator (AR-P) with more than five years of experience with the FEMD, at the same time of the day (±1 h) for each participant and under similar environmental conditions (∼21 °C and ∼60% humidity).

### 2.3. Isokinetic and Isometric Test

Before testing, participants completed a general warm-up consisting of 5 min of jogging, 5 min of joint mobility exercises, and a specific back-strength exercise using elastic bands. Once the warm-up was completed, participants proceeded to the test.

Participants completed two sets of four repetitions (one familiarization and one testing set) of a seated unilateral cable row at 0.30 m·s^−1^ using the FEMD coupled with a fixation system. Repetitions were performed consecutively without rest between them. Immediately after the dynamic repetitions, a single 6-s isometric contraction was performed in the final position (elbow flexed at 90°). This duration was selected to ensure a stable plateau of force production, following previous recommendations for reliability testing. A 3-min rest interval was provided between sets to minimize fatigue.

**Figure 1:**
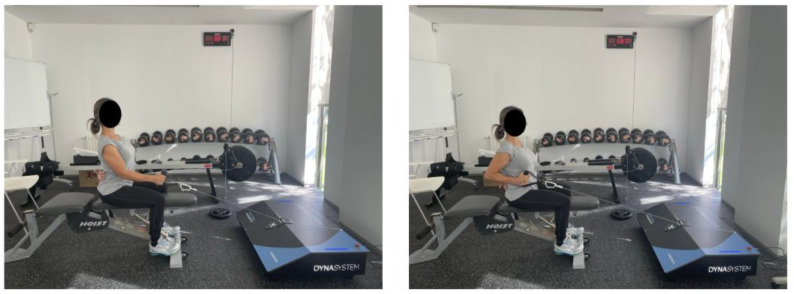
Initial position (left panel) and final position (right panel) of seated unilateral cable row exercise.

Participants were instructed to perform maximal voluntary contractions throughout all concentric and eccentric phases of the dynamic repetitions and during the isometric hold. The range of motion was individualized, beginning at full elbow extension (180°) measured with a 360° goniometer (Baseline Absolute Axis 360°, Fabrication Enterprises, Inc., White Plains, New York, USA) and ending at 90° of elbow flexion (Figure 1). These values were entered into the FEMD interface, corresponding to the measured cable displacement. Tests were conducted in a seated position (hips and knees flexed at 90°) on a bench (Hoist, Poway, CA, USA) placed 50 cm from the FEMD, with the non-testing hand placed behind the back.

### 2.4. Outcome Variables

For dynamic strength, the three highest repetitions were selected to compute peak and average force during concentric and eccentric contractions. For isometric strength, peak and average force values were obtained from the 6-s contraction.

### 2.5. Statistical Analyses

#### 2.5.1. Reliability

The data distribution was verified using the Shapiro–Wilk normality test. Reliability was analyzed through the intraclass correlation coefficient (ICC, model 3.1), coefficient of variation (CV), and standard error of measurement (SEM), each with 95% confidence intervals. The scale used for interpreting the magnitude of the effect size (ES) was specific to training research: negligible (<0.2), small (0.2–0.5), moderate (0.5–0.8), and large (≥0.8) [15]. The magnitude of the intraclass correlation coefficient values was determined using a qualitative scale: values close to 0.1 were considered low reliability; 0.3, moderate; 0.5, high; 0.7, very high; and values close to 0.9, extremely high. Bland–Altman analyses were performed to show the level of agreement between the tests and retests of the seated unilateral cable row exercise. The plots show that the bias and limits of agreement (LoA) were calculated to be 95%. Correlations between static and dynamic tests, as well as between dominant and non-dominant forces, were analyzed using Pearson’s correlation coefficient (*r*) for normally distributed variables and Spearman’s rank correlation coefficient (ρ) for non-normally distributed variables. Correlation magnitudes were interpreted as follows: small (0.10–0.29), moderate (0.30–0.49), large (0.50–0.69), very large (0.70–0.89), and extremely large (0.90–1.00).

A paired-sample *t*-test was used to determine the differences between the forces of the back muscles on the dominant and non-dominant sides. The level of significance was set at *p* < 0.05. To interpret the observed magnitude of differences in coefficients of variation of the dominant and non-dominant side and force manifestation, the mean of the CV was calculated and a default for the smallest important ratio of 1.15 was used [16]. Analyses were performed using a customized spreadsheet [17] and the SPSS software package (version 25.0).

#### 2.5.2. Asymmetries

Bilateral strength asymmetry was calculated as [(stronger arm − weaker arm)/stronger arm] × 100 and is expressed as a percentage. Positive values indicated stronger dominant-side muscles, whereas negative values indicated stronger non-dominant muscles [18]. Peak force values were used for asymmetry calculations in both isometric and dynamic conditions (concentric and eccentric). Additionally, the Bilateral Strength Asymmetry method was applied, as it has been shown to provide greater accuracy in the study of asymmetries with unilateral tests [19].

## 3. Results

**Figure 2:**
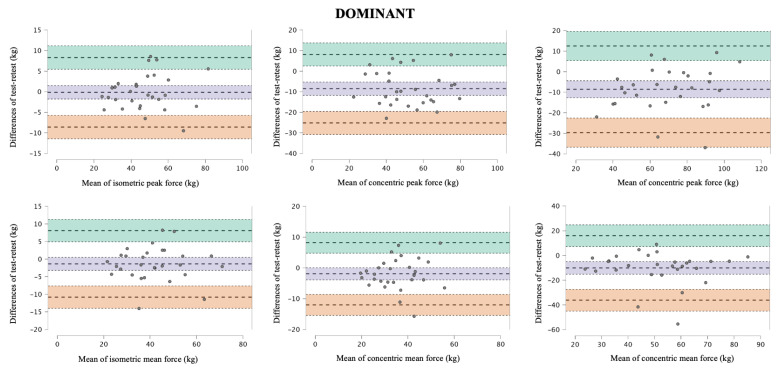
Bland–Altman plots for isometric, concentric, and eccentric peak and mean force measurements of the dominant seated unilateral cable row in the dominant limb. The central dashed line represents the mean difference (bias), the outer dashed lines represent the 95% limits of agreement, and the coloured dotted lines indicate the confidence intervals for both the mean difference (purple) and the concordance limits (green and orange) for the measurement of dominant seated unilateral cable row exercises between the test and retest.

**Figure 3:**
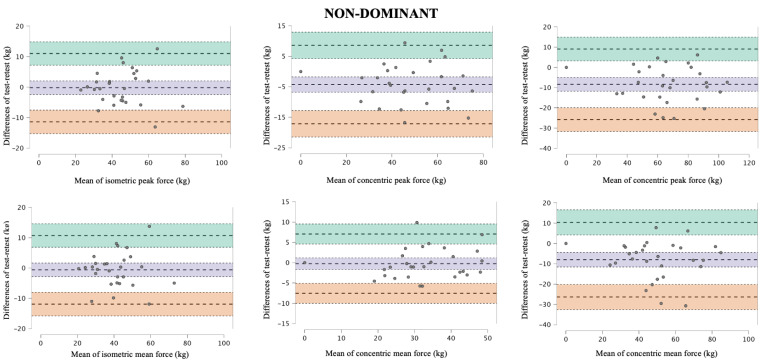
Bland–Altman plots for isometric, concentric, and eccentric peak and mean force measurements of the non-dominant seated unilateral cable row in the dominant limb. The central dashed line represents the mean difference (bias), the outer dashed lines represent the 95% limits of agreement, and the coloured dotted lines indicate the confidence intervals for both the mean difference (purple) and the concordance limits (green and orange) for the measurement of dominant seated unilateral cable row exercises between the test and retest.

**Figure 4:**
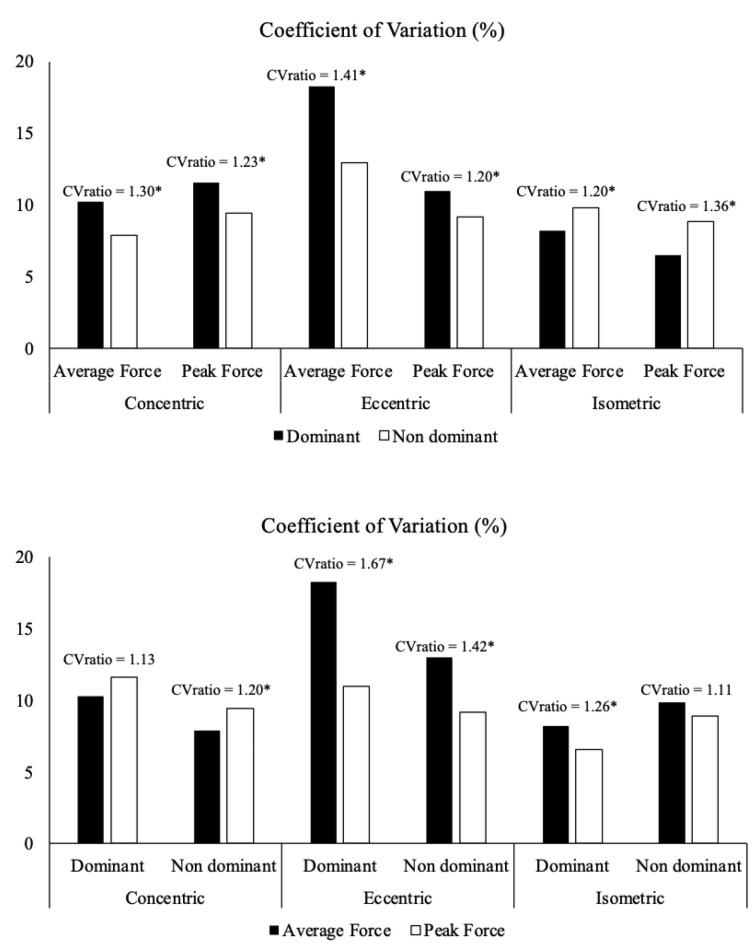
Comparison of the reliability of the outcomes of the dominant and non-dominant sides of the seated unilateral cable row exercise (upper panel) and the average and peak forces of the seated unilateral cable row exercise (lower panel). * Meaningful differences in reliability were identified as CVratio > 1.15. CV, coefficient of variation.

Table 1 shows the test and retest force data, as well as the absolute and relative reliability data for both dominant and non-dominant side back exercises. The peak and average force values had very high to extremely high relative reliability (ICC = 0.86–0.96) and acceptable absolute reliability (CV around 10%), except for the average eccentric phase force on the dominant side (CV = 18.27%; ICC = 0.72) (Table 1). Bland–Altman analyses for the dominant and non-dominant side revealed low mean biases and narrow limits of agreement across isometric, concentric, and eccentric conditions for both peak and mean force (Figure 2 and Figure 3). In addition, statistically significant differences in reliability were found between the dominant and non-dominant sides in all force assessments and all force manifestations, except for the dominant concentric phase and non-dominant isometric (CV ratio > 1.15) (Figure 4).

The correlation was performed with the retest data, considering the first session as a familiarization with the test. The correlations were very large and extremely large between the dominant and non-dominant sides, as well as between the dynamic and static force production of the back muscles (r = 0.76–0.95; *p* < 0.001) (Table 2).

**Figure 5:**
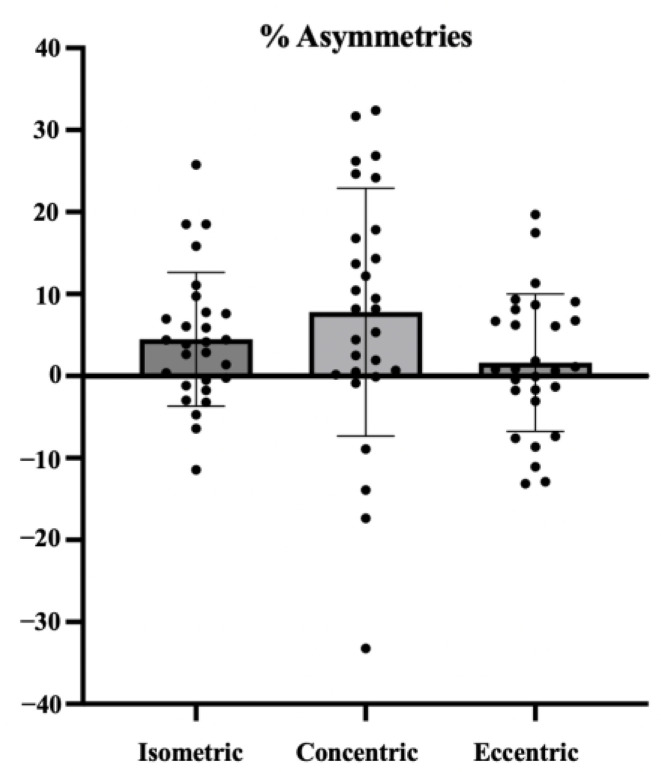
Percentages of asymmetries of everyone between the dominant and non-dominant sides in the isometric, concentric, and eccentric contractions of the seated unilateral cable row exercise and representation of the mean and standard deviation of the sample.

The retest was also used to study the differences in force between the dominant and non-dominant sides. Significant differences were found for the concentric peak force (*p* = 0.004), isometric force (*p* = 0.02), and concentric mean force (*p* = 0.001). No significant differences were observed between the dominant and non-dominant sides for the peak and average forces in the eccentric phase, nor for the isometric average force (*p* > 0.05). Related to interlimb asymmetries, in the isometric phase, an asymmetry of 4.47 ± 8.16% was found; in the concentric phase, 7.79 ± 15.01%; and in the eccentric phase, 1.63 ± 8.37%. When analyzing the data individually, 14%, 32%, and 7% of the subjects presented an asymmetry greater than 15% between the dominant and non-dominant sides in isometric, concentric, and eccentric forces, respectively (Figure 5).

## 4. Discussion

The present study aimed to analyze the reliability of the seated unilateral cable row exercise using an FEMD, to examine differences between contraction types and sides; investigate the relationship between the dominant and non-dominant sides, as well as between the dynamic and static force production of the back muscles; and to quantify interlimb asymmetries in back muscle force. The main findings were:(i) very high to extremely high relative reliability (ICC = 0.86–0.96) and acceptable absolute reliability for most force outcomes, with the exception of average eccentric force in the dominant side; (ii) very large to extremely large correlations between dominant and non-dominant sides, as well as between dynamic and isometric conditions; and (iii) significant differences in force output between sides, with moderate levels of interlimb asymmetry, particularly in the concentric phase.

The high reliability values observed in this study are consistent with previous reports demonstrating that electromechanical dynamometers provide stable measures of force across multiple contraction types and muscle groups [10,20,21,22]. Our findings extend this evidence to a multi-joint, unilateral pulling exercise that better reflects sport-specific and rehabilitation contexts compared to isolated isokinetic tests. Notably, eccentric average force in the dominant side showed greater variability (CV = 18.27%; ICC = 0.72), likely due to the technical complexity of eccentric control and the need for greater familiarization [20,23,24]. Reliability was generally higher in the dominant limb for both mean and peak force, except under isometric conditions. Across contraction types, mean force proved more reliable in concentric actions, whereas peak force demonstrated higher reliability in eccentric and isometric assessments. These patterns align with Mula-Pérez et al. [25], who reported superior intersession reliability for the dominant side in isometric evaluations.

In the comparison between dominant and non-dominant sides, significant differences were found in concentric average and peak forces, as well as in isometric peak force. These results are consistent with previous research on volleyball players, which showed significant differences in shoulder internal rotation strength between sides during the concentric phase [26]. Another study on football players using a flywheel device revealed significant differences in leg curl and leg extension power between sides [27]. These findings suggest that the sample may be accustomed to resistance training with a strong concentric emphasis, explaining the observed differences in this phase. Additionally, in young elite soccer players, spinal asymmetries have been linked to alterations in specific fitness parameters, supporting the view that side-to-side differences may be task- and sport-specific and should be interpreted at the individual level [28]. In contrast, no significant differences emerged in the eccentric phase, although evidence of a learning effect was present.

While the literature on asymmetries is extensive, most studies focus on the lower limbs and assess concentric or isometric phases in football or soccer athletes [27,29,30], making comparisons with eccentric data more difficult. Moreover, asymmetries appear task-dependent; after unilateral trunk tasks, side-specific thermal responses have been observed, underscoring the value of contraction-mode– and task-specific protocols [31]. Consistent with prior syntheses, the asymmetry–performance relationship is heterogeneous and task dependent, which justifies an individual-level interpretation rather than overarching generalizations [30,32]. Injury risk thresholds have been established at 10–15% asymmetry [18]. Although group averages fell below this threshold, individual analysis revealed that three participants exceeded 15% asymmetry in both isometric and concentric force, one participant exceeded it only in the isometric phase, five in the concentric phase, and two in the eccentric phase (who also showed asymmetry in the concentric phase). These findings underscore the importance of analyzing individual asymmetry values when assessing injury risk in athletes.

This study is not without limitations. The sample consisted of young, physically active athletes rather than athletes from a specific sport, limiting the generalizability of the findings. Nonetheless, the data are valuable for demonstrating the reliability of the test and its potential for assessing asymmetries in athletic populations. Furthermore, the asymmetry data could not be directly compared with previous studies due to the lack of unilateral assessments of back muscles. The most innovative aspect of this study lies in its unilateral evaluation of maximum isometric and isokinetic back muscle strength to investigate asymmetry. Additionally, asymmetries were analyzed individually and categorized by contraction type—an approach not commonly found in the literature, which typically focuses on performance metrics (e.g., jumping, sprinting) and lower limb strength.

## 5. Conclusions

In conclusion, the seated unilateral cable row exercise assessed with an FEMD demonstrates very high reliability across most contraction types and force measures. The method is sensitive enough to detect interlimb differences and asymmetries, which appear to be most pronounced in concentric conditions. These results support the application of unilateral back muscle assessments in both athletic and rehabilitative settings, where monitoring of side-to-side imbalances is essential for performance optimization and injury prevention.

## 6. Practical Applications

Coaches and medical professionals can apply this test to reliably assess unilateral force in back muscle exercises among young, physically active athletes, placing particular emphasis on peak isometric force in the dominant arm. As a unilateral assessment, it allows coaches and physical trainers to identify asymmetries in these young athletes—an essential consideration for both performance optimization and injury prevention. By quantifying the force output of each upper limb, practitioners can establish reference values across the competitive season and use them as a foundation for designing individualized training and rehabilitation programs.

## Figures and Tables

**Table 1:** Absolute and relative test–retest reliability of the mean and peak force in dominant and non-dominant seated unilateral cable row exercise.

		Test (kg)	Retest (kg)	*p*-Value	ES	CV (95% CI)	SEM (95% CI)	ICC (95% CI)
**Dominant**								
Average Force								
Isokinetic	Con	34.6 (10.3)	36.5 (9.9)	0.07	0.18	10.25 (8.13–13.86)	3.64 (2.89–4.93)	0.88 (0.76–0.94)
	Ecc	46.2 (16.7)	56.7 (17.6)	0.001	0.61	18.27 (14.50–24.71)	9.40 (7.46–12.72)	0.72 (0.48–0.86)
Isometric		40.2 (13.1)	41.8 (12.9)	0.07	0.12	8.22 (6.57–10.98)	3.37 (2.69–4.50)	0.94 (0.87–0.97)
Peak Force								
Isokinetic	Con	47.5 (15.7)	56.1 (16.3)	0.001	0.53	11.59 (9.20–15.67)	6.00 (4.76–8.12)	0.87 (0.74–0.94)
	Ecc	65.2 (22.1)	73.7 (20.0)	0.001	0.41	10.96 (8.70–14.83)	7.61 (6.04–10.30)	0.88 (0.76–0.94)
Isometric		45.9 (14.4)	46.1 (14.3)	0.77	0.02	6.54 (5.22–8.74)	3.00 (2.40–4.02)	0.96 (0.92–0.98)
**Non-dominant**								
Average Force								
Isokinetic	Con	33.9 (9.6)	34.2 (8.8)	0.77	0.02	7.88 (6.23–10.73)	2.68 (2.12–3.65)	0.92 (0.84–0.96)
	Ecc	47.2 (17.2)	55.5 (17.3)	0.001	0.48	12.98 (10.26–17.66)	6.66 (5.27–9.07)	0.86 (0.72–0.93)
Isometric		39.8 (12.2)	40.4 (12.0)	0.60	0.05	9.83 (7.83–13.33)	3.95 (3.14–5.30)	0.90 (0.80–0.95)
Peak Force								
Isokinetic	Con	47.5 (14.7)	51.9 (14.9)	0.001	0.30	9.43 (7.46–12.84)	4.69 (3.71–6.38)	0.91 (0.81–0.96)
	Ecc	64.5 (19.8)	73.2 (19.6)	0.001	0.44	9.15 (7.24–12.46)	6.30 (4.98–8.58)	0.90 (0.80–0.95)
Isometric		43.9 (12.9)	44.2 (13.0)	0.79	0.02	8.87 (7.06–11.92)	3.91 (3.11–5.25)	0.91 (0.83–0.96)

Con = concentric contraction; Ecc = eccentric contraction; ES = effect size; CV = coefficient of variation; SEM = standard error of measurement (kg); ICC = intraclass correlation coefficient; 95% CI = 95% confidence interval. Data are presented as mean (standard deviation).

**Table 2:** Correlation between dominant and non-dominant side and dynamic with static force of seated unilateral cable row exercise.

Dominant with Non-Dominant		
Peak Force	Concentric	*r* = 0.83 ***
	Eccentric	ρ = 0.93 ***
	Isometric	ρ = 0.90 ***
Average Force	Concentric	ρ = 0.94 ***
	Eccentric	ρ = 0.94 ***
	Isometric	ρ = 0.90 ***
**Dynamic with static force**		
Peak Force	Concentric-Isometric Dominant	ρ = 0.89 ***
	Eccentric-Isometric Dominant	ρ = 0.93 ***
	Concentric-Isometric Non-Dominant	*r* = 0.76 ***
	Eccentric-Isometric Non-Dominant	*r* = 0.88 ***
Average Force	Concentric-Isometric Dominant	*r* = 0.88 ***
	Eccentric-Isometric Dominant	ρ = 0.95 ***
	Concentric-Isometric Non-Dominant	ρ = 0.80 ***
	Eccentric-Isometric Non-Dominant	ρ = 0.88 ***

Pearson’s correlation coefficient (*r*) was used for normally distributed variables, whereas Spearman’s rank correlation coefficient (ρ) was used for non-normally distributed variables. Significance level *** *p* < 0.001.

## Data Availability

Data supporting the reported results can be requested directly at evila@uvigo.es.
